# The clinical value of indirect immunofluorescence for screening anti-rods and rings antibodies: A retrospective study of two centers in China

**DOI:** 10.3389/fimmu.2022.1007257

**Published:** 2022-09-27

**Authors:** Jingjing Meng, Guoxiang Yang, Siting Li, Yueming Luo, Yina Bai, Chuiwen Deng, Ning Song, Mengtao Li, Xiaofeng Zeng, Chaojun Hu

**Affiliations:** ^1^ Department of Rheumatology and Clinical Immunology, Chinese Academy of Medical Sciences & Peking Union Medical College, National Clinical Research Center for Dermatologic and Immunologic Diseases (NCRC-DID), Ministry of Science & Technology, Key Laboratory of Rheumatology & Clinical Immunology, Ministry of Education, Beijing, China; ^2^ Department of Clinical Laboratory, Fifth Affiliated Hospital of Zhengzhou University, Zhengzhou, China; ^3^ Department of Clinical Laboratory, Inner Mongolia People’s Hospital, Hohhot, China; ^4^ Jiangmen Wuyi Hospital of Traditional Chinese Medicine (TCM) (Affiliated Jiangmen TCM Hospital of Ji’nan University), Jiangmen, China

**Keywords:** anti‐rods/rings(anti-RR), anti-nuclear antibody (ANA), hepatitis C, autoimmune diseases (AIDs), prevalence

## Abstract

**Objective:**

To investigate the distribution and clinical significance of the rods and rings (RR) pattern in various diseases.

**Methods:**

A total of 169,891 patients in Peking Union Medical College Hospital (PUMCH) and 29,458 patients in Inner Mongolia People’s Hospital (IMPH) from January 2018 to December 2020 were included, and the results of ANA (antinuclear antibodies) and special antibodies were analyzed retrospectively.

**Results:**

The positive rates of ANA and RR patterns were 34.84%, 0.16% in PUMCH, and 44.73%, 0.23% in IMPH. Anti-RR antibodies mainly appear in adults (≥ 41 years), mostly of low or medium fluorescence titers. Isolated RR patterns were mostly presented (60.30% and 69.12%, respectively), and the RR pattern mixed with the speckled pattern was most commonly observed among patients having two or more patterns. The RR pattern existed in a variety of diseases including hepatitis C, AIDs, pulmonary diseases, nephropathy diseases, and even healthy people. The highest prevalence of the RR pattern was observed in hepatic diseases, such as hepatic dysfunction (0.79%), hepatic cirrhosis (1.05%), PBC (0.85%), and AIH (0.65%), etc. The positive rate of specific antibodies in RR pattern cases was 31.25%, and anti-Ro52 (27, 20.61%) was the most common target antibody.

**Conclusion:**

The RR pattern had a low prevalence in ANAs test samples and varied in different nationalities and regions. Except for hepatitis C, it could be observed in AIDs, pulmonary diseases, nephropathy, other hepatic diseases, and even healthy people, but the positive rate was slightly higher in hepatic diseases. Its mechanism of action and clinical relevance still need clarification.

## Introduction

Antinuclear antibodies (ANAs) represent a class of autoantibodies against cellular components, including nuclear, cytoplasmic, cytoskeleton, and cyclin. It can be found in most autoimmune diseases (AIDs) (1), and plays an important role in the diagnosis, classification, treatment, and disease activity monitoring of AIDs (2, 3), such as Sjogren’s syndrome (SS), systemic lupus erythematosus(SLE), and dermatomyositis (DM), etc. (4–7) As well as, it is also detected in cancers, chronic infectious diseases(such as viral infections, tuberculosis, bacterial endocarditis, etc), medication-related adverse events, and even healthy individuals (4, 8–10). While the determination of ANA by indirect immunofluorescence (IIF) using human epithelial type 2 (HEp-2) cell substrate will provide more information about the disease state and types through fluorescence intensity and patterns. For instance, in most cases, higher fluorescence intensity indicates a higher antibody level, which is better correlated with ANA related rheumatic disease (AARD) (11).

According to the International Consensus on ANA Patterns (ICAP), ANA patterns are classified by the anti-cell (AC) code including 29 patterns (from AC-01 to AC-29), which must be reported (12). The “rods and rings” (RR) pattern, first discovered in the serum of patients with chronic hepatitis C in 2005, corresponds to the AC‐23 pattern. Fluorescence of RR is characterized by 3-10 µm long rods and 2-5 µm diameter rings in the cytoplasm of interphase cells, with some smaller rods and rings also appearing in the nucleus during the division stage (13–17). Some studies show that inosine 5′‐monophosphate dehydrogenase 2 (IMPDH2) and cytidine 5′‐triphosphate synthase 1 (CTPS1) are all located in RR structures. The target antigen of anti‐RR is conserved intracellular polymeric structures which are the composition of IMPDH2 induced by IMPDH2 inhibitors/glutamine deprivation condition (18, 19). IMPDH2 could be inhibited by retroviral agent ribavirin (RBV) related to the Hepatitis C virus (HCV) treatment. In the serums of HCV patients receiving interferon-α (IFN) and RBV combined treatment (IFN/RBV), RBV shows the ability to induce intracellular structure *in vitro*, which was also the cause of the anti-RR immune response (20, 21). Nevertheless, it is reported that a few subjects presenting anti-RR autoantibodies only had a positive history of methotrexate and acyclovir treatment without the history of HCV and IFN/RBV treatment (22). In addition, positive anti-RR also appears occasionally in individuals with other diseases and even a paucity of healthy persons (0.8%) (23–26).

All in all, the distribution and clinical significance of the RR pattern in healthy populations and patients with different diseases are not well studied. Therefore, in the study, results of 199,349 cases with ANA IIF from two centers were analyzed retrospectively to clarify the characteristics of the RR pattern between different diseases and populations to further explore its clinical significance.

## Materials and methods

### Patients

From January 2018 to December 2020, 169,891 patients (excluding duplicates) from the Peking Union Medical College Hospital (PUMCH) and 29,458 patients (excluding duplicates) from Mongolia People’s Hospital (IMPH) were sequentially included in this study. All them underwent ANA IIF tests and the data were retrospectively analyzed. The study was conducted following relevant guidelines and regulations, and also obtained the ethical approval and consent of the hospital ethics committee. Other clinical and laboratory information such as disease diagnosis and routine blood test results were collected from the information system of each hospital.

### ANA detection

Both centers performed ANA detection following the manufacturer’s protocols (Euroimmun, Germany) indirect immunofluorescence (IIF) using HEp-2 cell substrate and primate liver tissue. The serums were performed at a 1:100 dilution, incubated with the substrate slides for 30 min, and washed for 5min. Then the fluorescein isothiocyanate anti-human IgG conjugate was added and incubated for 30 min in light protection, and subsequentially washed for 5min. The substrate slides were mounted with glycerol, and slides determining ANA were read by two experienced technologists under a fluorescence microscope (Olympus BX51). The ANA titer of more than 1:80 (≥1:80) was considered a positive result in this study. The interpretation of the ANA test was done by two observers experienced in pattern reading. Samples displaying the RR pattern were determined according to pattern-related characteristics.

### Line immunoblot assay

To explore the potential association with other autoantibodies, partial sera positive for anti-RR by LIA were further screened for fifteen autoantibodies (nRNP/Sm, Sm, SSA/Ro60, Ro52/TRIM21, SSB/La, Scl-70, PM-Scl, Jo-1, CENP-B, PCNA, dsDNA, nucleosomes, histones, ribosomal P protein (Rib-P), AMA-M2) using the Euroline ANA Profile 3 (Euroimmun, Germany) according to the manufacturer’s instructions (27).

### Chemiluminescent immunoassay assay

Anti-AMA-M2, anti-GP210, anti-SP100, anti-SLA CLIA kits were obtained from HOB Biotech Group (China) (BioCLIA Autoimmune), according to the manufacturer’s instructions described in the assay procedure. The assay was performed on SMART 6500 instrument (Chongqing Keysmile Biological Technology Co., Ltd.).

### Statistical analysis

All dates were analyzed using Statistical Package for Social Sciences (SPSS) (IBM SPSS Statistics 19). Continuous variables of non-normal distribution such as age were presented as median with the interquartile range. The Chi-square test and the Fisher exact test when appropriate were used to analyze unordered categorical variables, such as sex, age group, and various positive rates, and the Mann Whitney U test was used for comparing age and ANA–HEp-2 titers between groups. P < 0.05 was considered statistically significant.

## Results

### The demographic characteristics of RR and ANA-positive patients

The demographic characteristics of patients from PUMCH and IMPH were shown in **Supplementary Table S1**. Patients from PUMCH ranged from 1 to 107 years old with a median age of 44(31-56) years, of which 56,660 were males with a median age of 46(32-58) years and 113,231 were females with a median age of 43(31-55) years. Patients from IMPH ranged from 0 to 105 years old with a median age of 54(39-65) years, of which 10,783 were males with a median age of 57(42-68) years and 18,675 were females with a median age of 53(38-64) years. The demographic characteristics of RR and ANA-positive patients from two centers were presented in **Table 1**. The prevalence of RR and ANA in PUMCH were significantly lower than that in IMPH, but the RR pattern positive rate in HEp-2 IFA positive sera showed no statistical difference. There were more female compared to male patients in both the RR positive and ANA positive group (P<0.01) in the two centers. A significant difference was observed in the gender ratio in ANA positive between the two centers, but not in RR-positive patients. At the same time, the proportion of female patients in PUMCH was more than that in IMPH. In every age group, the median age of ANA positive in the IMPH was higher than that in the PUMCH (P<0.05), except for the age group less or equal to 20 years. However, there was no significant difference in the median age of RR positive between in two centers, except for the median age of overall patients. In the PUMCH, ANA positive patients aged 41 to 60 years were the most, accounting for 38.1%, as well as patients with the RR pattern aged 41 to 60 years and 61 years and above were the most, accounting for 36.3% and 34.5%. In the IMPH, patients with ANA positive or RR positive aged 61 years and above were the most, accounting for 44.2% and 60.3%.

**Table 1:** The demographic characteristics of RR and ANA-positive patients from two centers.

Variables	RR pattern			ANA positive		
	PUMCH	IMPH	*P_1_ value*	PUMCH	IMPH	*P_2_ value*
Case (%^*^)	267 (0.16%)	68 (0.23%)	0.004	59182 (34.84%)	13178 (44.73%)	0.000
M: F	1:1.3	1: 1.5	0.656	1: 4.5	1: 2.4	0.000
Median age	54 (38-64)	64 (53-71)	0.000	46 (32-58)	58 (46-68)	0.000
Distribution of age (Median age, %^**^)						
≦20	17 (13-19), 7.5% ^a^	10 (1-.), 2.9% ^a*^	0.623	15 (11-18), 7.8% ^a^	13 (8-18), 2.6%^a*^	0.000
21-40	32 (27-37), 21.7% ^b^	35 (31-39), 10.3% ^a*^, ^b*^	0.408	31 (29-36), 33.7% ^b^	32 (28-36), 16.5% ^b*^	0.000
41-60	52 (47-57), 36.3% ^c^	55 (51-56), 26.5% ^b*^	0.227	51 (46-55), 38.1% ^c^	52 (47-56), 36.7% ^c*^	0.000
≧61	67 (64-72), 34.5% ^c^	70 (65-78), 60.3% ^c*^	0.055	67 (64-72), 20.4% ^d^	66 (63-71), 44.2% ^d*^	0.000

PUMCH, Peking Union Medical College Hospital. IMPH, Inner Mongolia People’s Hospital; ANA, antinuclear antibodies; RR, rods and rings; M, Male; F, Female..The “%^*^” indicated the percentage of positive cases to total cases in respective hospitals. The “%^**^” indicated the percentage of positive cases in this age group to the total positive cases in respective hospitals. The “P_1_ value “ and “P_2_ value “indicated the comparison of results between the two hospitals. The “a”, “b”, “c”, “d” and “a^*^”, “b^*^”, “c^*^”, “d^*^” in the column indicated the comparison of results among various age groups in respective hospitals. Therefore, the Symbol “*” and letters which are as a whole represent the comparison results in IMPH.

### Analysis of the immunofluorescent pattern of the RR pattern

**Figure 1:**
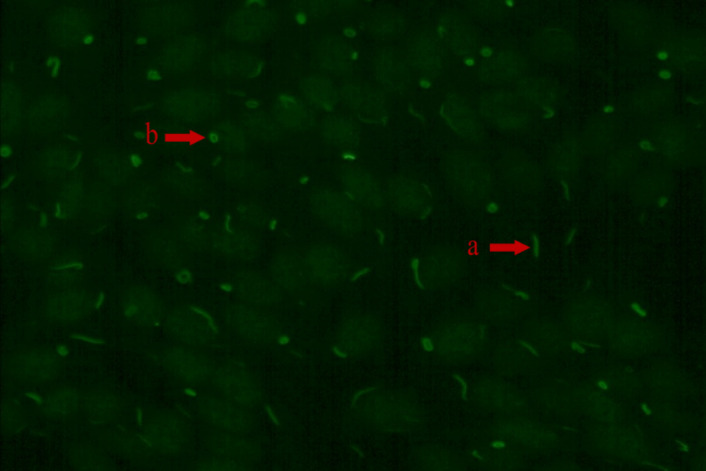
The RR fluorescence pattern (IIF, x200) “a” arrow points to rod and “b” arrow point to ring.

**Figure 2:**
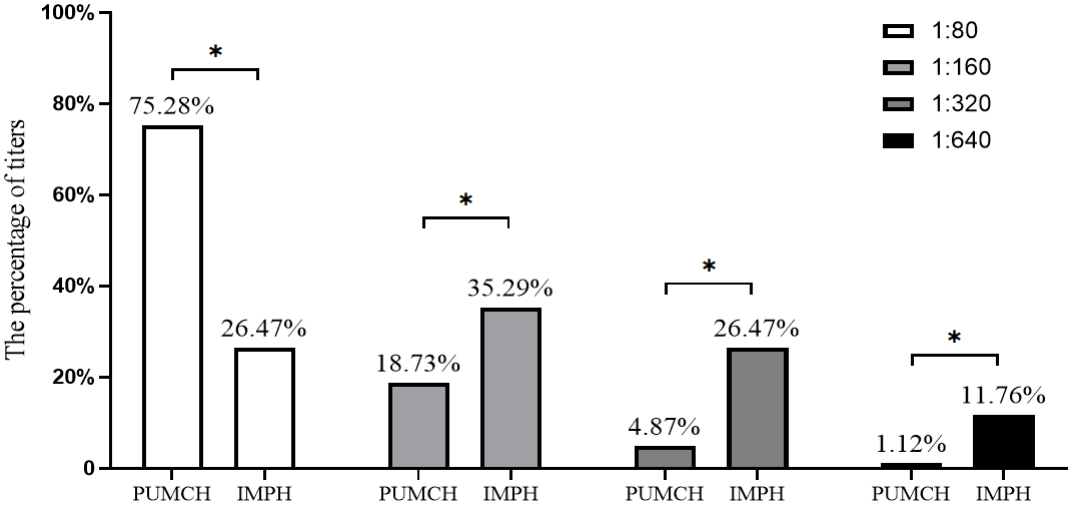
The percentage comparison of each titer in RR pattern cases between different centers. PUMCH, Peking Union Medical College Hospital. IMPH, Inner Mongolia People’s Hospital. The percentage above the bars indicates the percentage of each titer in RR pattern cases. “*” indicated the percentage comparison of each titer in RR pattern cases between different centers is statistically significant.

In the study, the fluorescence pattern of patients with positive anti-RR antibodies was shown in **Figure 1**. A significant difference was observed in the distribution of titers for the RR pattern in the two centers (p<0.05, **Figure 2**). The proportion of the 1:80 low titer group in PUMCH was much higher than that in IMPH. Among 267 cases of the RR pattern in PUCMH, the low titer of 1:80 was the most common (201, 75.28%), followed by a medium-high titer of 1:160 (50, 18.73%) and 1:640 of high titer (3, 1.12%). Among 68 cases in IMPH, 1:640 of high titer was the least (8, 11.76%), and the proportion of other titer groups was similar (p>0.05). As shown in **Table 2**, fluorescence patterns of patients with RR patterns in two centers were mainly isolated RR patterns. Among patients having two or more patterns, the speckled pattern was found more frequently than other fluorescence patterns. The speckle pattern accounted for 49.06% (52/106) in PUMCH and 90.48% (19/21) in IMPH. The mixed patterns include only the speckled pattern (106 in PUMCH, 21 in IMPH), the mixture of the cytoplasmic and speckled pattern (4 in PUMCH, 4 in IMPH), the mixture of the nucleolar and speckled pattern (4 in PUMCH, 1 in IMPH), the mixture of the homogeneous and speckled pattern (3, 0), and the mixture of spindle fibers and speckled pattern (2 in PUMCH, 0 in IMPH). Moreover, the median of complex RR pattern titer in PUMCH was relatively low (mainly 1:80 in PUMCH compared to above or equal to 1:320 in IMPH).

**Table 2:** Fluorescence patterns of RR-positive patients.

Fluorescence patterns		PUMCH			IMPH		
		Patients	Range of titers	Median titer	Patients	Range of titers	Median titer
**RR**		161 (60.3%)	1:80-1:640	1:80	47 (69.12%)	1:80-1:640	1:160
**Mixed pattern**		106 (39.7%)	1:80-1:640	1:80	21 (30.43%)	1:80-1:640	1:320
1	RR-Speckled	39 (14.61%)	1:80-1:320	1:80	14 (20.29%)	1:80-1:640	1:320
2	RR-Homogeneous	26 (9.74%)	1:80-1:640	1:80	−	−	−
3	RR-Cytoplasmic	18 (6.74%)	1:80-1:640	1:80	1 (1.45%)	1:320	1:320
4	RR-Nucleolar	2 (0.75%)	1:80	1:80	−	−	−
5	RR-Membranous	2 (0.75%)	1:80-1:160	−	−	−	−
6	RR-Centromere	5 (1.87%)	1:80-1:320	1:80	1 (1.45%)	1:320	1:320
7	RR-Speckled-Cytoplasmic	4 (1.5%)	1:80-1:320	1:80	4 (5.8%)	1:160-1:640	1:480^*^
8	RR-Speckled-Nucleolar	4 (1.5%)	1:80	1:80	1 (1.45%)	1:640	1:640
9	RR-Speckled-Homogeneous	3 (1.12%)	1:80-1:160	1:80	−	−	−
10	RR-Speckled-Spindle fibers	2 (0.75%)	1:80-1:160	−	−	−	−
11	RR-Homogeneous-Cytoplasmic	1 (0.37%)	1:160	1:160	−	−	−

PUMCH, Peking Union Medical College Hospital; IMPH, Inner Mongolia People’s Hospital. “*” indicated the median value is calculated.

### The distribution of diseases in the patients with RR pattern and ANA positive

**Table 3** and **Supplementary Table S2** illustrated the disease distribution of the RR pattern and ANA-positive (excluding RR) in two centers. In ANA-positive cases, AIDs, pulmonary diseases, nephropathy diseases, and dermatosis were most common in the PUMCH, while in IMPH the top three diseases were nephropathy diseases, AIDs, and arthropathy. Among 267 patients with RR patterns in PUMCH, AID (19.48%) was the most, mainly presented as complex RR patterns, followed by pulmonary diseases (16.48%), and nephropathy diseases (9.74%). However, among 68 patients with RR pattern in IMPH, nephropathy diseases (26.47%), pulmonary diseases (16.18%), and AIDs (7.35%) ranked as the three most frequent diagnoses. In pulmonary diseases, it appeared the isolated RR pattern was the main, whereas no diffidence between patterns was observed in other diseases. Among all the physical examination patients with the RR pattern, isolated RR was the most common. In RR pattern cases, the proportion of hepatic disease was only 5.24% in PUMCH and 2.94% in IMPH.

**Table 3:** The distribution of diseases in the patients with RR pattern and ANA positive.

Diseases	PUMCH			IMPH			*P*
	Isolated	Complex	Total (n, %^*^)	Isolated	Complex	Total (n, %^*^)	
**Autoimmune diseases**	19	32	52 (19.48%)	3	2	5 (7.35%)	0.018
CTD	2	11	13 (4.87%)	0	0	0	**−**
RA	7	5	12 (4.49%)	2	1	3 (4.41%)	1.000
SSc	4	4	8 (3.00%)	0	0	0	**−**
PBC	2	2	4 (1.50%)	0	0	0	**−**
AIH	0	1	1 (0.37%)	0	0	0	**−**
APS	0	1	1 (0.37%)	0	0	0	**−**
SLE	1	3	4 (1.50%)	0	1	1 (1.47%)	1.000
Systemic vasculitis	3	1	4 (1.50%)	1	0	1 (1.47%)	1.000
SS	0	4	4 (1.50%)	0	0	0	**−**
UC	0	1	1 (0.37%)	0	0	0	**−**
**Pulmonary diseases**	19	7	44 (16.48%)	17	1	11 (16.18%)	0.952
Pulmonary interstitial diseases	6	1	23 (8.61%)	5	0	2 (2.94%)	0.112
Neoplasm	4	1	6 (2.25%%)	1	0	1 (1.47%)	1.000
Infection	4	1	5 (1.87%)	2	0	1 (1.47%)	1.000
Others*	5	4	9 (3.375)	9	1	7 (10.29%)	0.026
**Nephropathy**	19	25	26 (9.74%)	9	3	18 (26.47%)	0.000
Renal insufficiency	9	14	7 (2.62%)	2	0	5 (7.35%)	0.061
Renal failure	3	2	6 (2.25%)	0	1	3 (4.41%)	0.394
Proteinuria	5	5	5 (1.87%)	0	1	2 (2.94%)	0.598
Others*	2	4	8 (3.00%)	7	1	8 (11.76%)	0.002
**Hepatic diseases**	10	4	14 (5.24%)	1	1	2 (2.94%)	0.541
Hepatic dysfunction	7	3	10 (3.74%)	0	0	0	**−**
Hepatic cirrhosis	2	0	2 (0.75%)	0	1	1 (1.47%)	0.459
Hepatitis B	1	0	1 (0.37%)	0	0	0	**−**
Hepatitis C	0	0	0	1	0	1 (1.47%)	**−**
Hepatic tumor or cancer	0	1	1 (0.37%)	0	0	0	**−**
**Other diseases**							
Endocrine disease*	15	4	19 (7.12%)	3	1	4 (5.88%)	0.719
Dermatosis*	15	5	20 (7.49%)	0	0	0	**−**
Arthropathy*	8	3	11 (4.12%)	4	1	5 (7.35%)	0.264
Hematological diseases*	7	8	15 (5.62%)	2	2	4 (5.88%)	1.000
Fever	8	5	13 (4.87%)	1	0	1 (1.47%)	0.211
Undefined diseases*	5	4	8 (3.00%)	6	4	10 (14.71%)	0.000
**Health examination**	22	5	27 (10.11%)	0	0	0	**−**

PUMCH, Peking Union Medical College Hospital; IMPH, Inner Mongolia People’s Hospital; CTD, connective tissue disease; RA, rheumatoid arthritis; SSc, systemic sclerosis; PBC, primary biliary cirrhosis; AIH, autoimmune hepatitis; APS, antiphospholipid syndrome; SLE, systemic lupus erythematosus; SS, Sjogren’s syndrome; UC, ulcerative colitis; The “%^*^” indicated the percentage of positive cases to total cases in respective hospitals. The diseases included in the disease * will be described in **Supplementary Table S2**. Some cases with the RR pattern suffered from more than one kind of disease simultaneously and were included in the analysis of some diseases repetitively.

**Figure 3:**
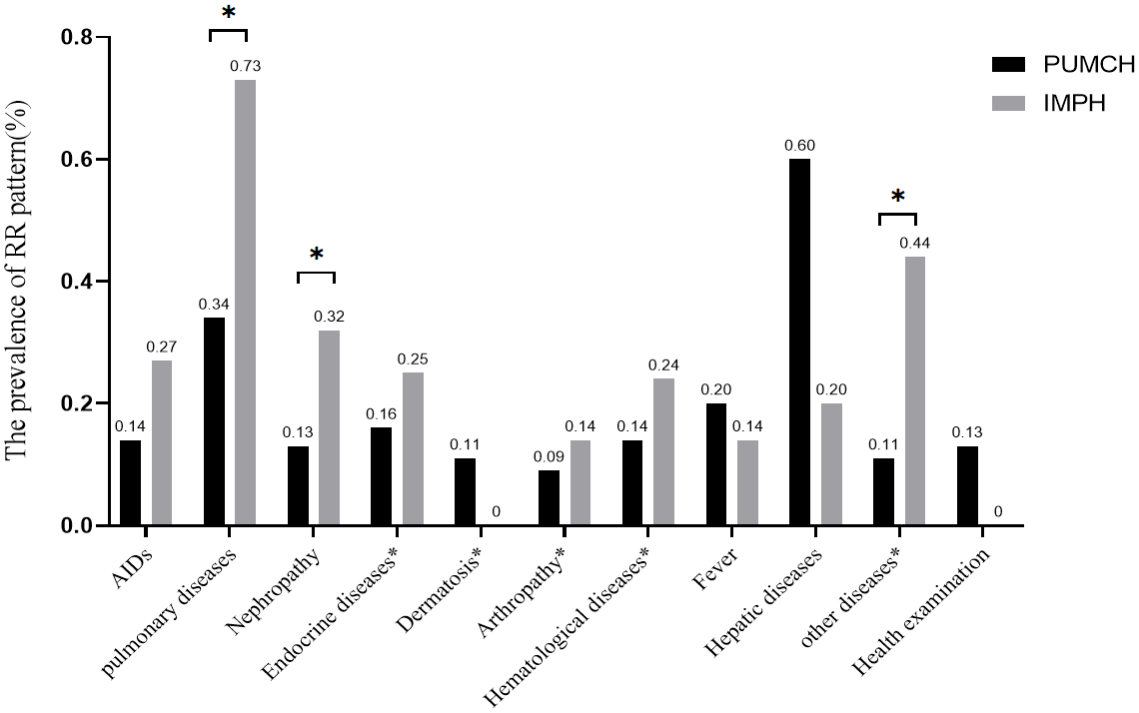
The prevalence of the RR pattern in different diseases. The percentage above the bars indicates RR positive cases of this disease to total RR positivity cases in each center. AIDSs included CTD, RA, SSc, PBC, AIH, APS, SLE, Systemic vasculitis, SS, and UC. Pulmonary diseases included pulmonary interstitial diseases, neoplasm, infection, and others". Nephropathy included renal insufficiency, renal failure, proteinuria, and others”. The diseases included in the disease * will be described in **Supplementary Table S2**. “*” means that the difference between them is statistically significant.

As shown in **Figure 3** and **Supplementary Table S3**, the prevalence of RR pattern was the highest in hepatic disease in PUMCH, but there was no difference with pulmonary diseases. Moreover, the prevalence of the RR pattern was higher in PBC and AIH than that in other AIDs, but only the difference between PBC and other AIDs except AIH and UC was statistically significant. And though it was the highest in pulmonary diseases based on dates from IMPH, there was no difference among diseases.

### Prevalence of the special antibody and clinical relevance in patients with RR pattern

In all RR pattern cases, only 128 cases had tested the ANA spectrum, including 69 isolated and 59 complex respective. Of the 64 cases tested for ASMA, 39 were isolated and 25 were complex respective. Of the 64 cases tested for AMA, 41 were isolated and 23 were complex respective. Of the 47 cases tested for AMA-M2, 31 were isolated and 23 were complex respective. Of the 64 cases tested for AMA, 41 were isolated and 16 were complex respective. Of the 18 cases tested for GP210, 8 were isolated and 10 were complex respective. Of the 18 cases tested for SP100, 8 were isolated and 10 were complex respective. Of the 39 cases tested for SLA, 16 were isolated and 23 were complex respective. Of 128 cases tested ANA spectrum, 40(31.25%) cases had the positive special antibody (in **Table 4**). Ro52 (27, 21.09%) and SSA (14, 10.94%) were the most common target antigens in the ANA spectrum. Among patients with Ro52 positive, there were 6 cases with ILD, 4 cases with SS, 3 cases with PBC, etc. Among the autoantibodies related to autoimmune liver disease, the antibody with the highest positive rate was AMA-M_2_(CLIA). And in these patients, it included 6 cases of PBC, 2 cases of pulmonary neoplasms, etc.

**Table 4:** Analysis of target antigen corresponding to RR pattern.

Specific antibody	Total	Antibody positive (n, %)			Diagnosed disease
		Isolated	Complex	Total	
**ANA spectrum**					
RNP/Sm	128	1	10	11 (8.59%)	CTD, SLE, APS, SSc, etc.
Sm	128	0	5	5 (3.91%)	CTD, SLE, APS, etc.
SSA	128	2	12	14 (10.94%)	CTD, SLE, APS, SS, PBC, interstitial pneumonia, etc.
Ro 52	128	6	21	27 (21.09%)	CTD, SLE, SS, interstitial pneumonia, pancytopenia, etc.
SSB	128	0	3	3 (2.34%)	SS, pancytopenia
Scl-70	128	2	1	3 (2.34%)	Localized scleroderma, fever, contracture of metacarpal fascia
PM-Scl	128	0	0	0	−
Jo-1	128	2	0	2 (1.56%)	arthrosis, enlargement of parotid gland
CENP-B	128	0	2	2 (1.56%)	RA, pulmonary infection
PCNA	128	0	0	0	−
Nucleosomes	128	2	1	3 (2.34%)	SLE, hyperlipidemia
Histone	128	1	1	2 (1.56%)	SLE, subcutaneous nodule
Rib-P	128	0	4	4 (3.13%)	SLE, APS, GBS, ascites
AMA-M_2_	128	2	4	6 (4.69%)	SS, PBC, HA
**Others**					
ASMA^*^	64	0	1	1 (1.56%)	Pulmonary space-occupying lesion
AMA^*^	64	3	5	8 (12.5%)	SS, PBC
$\text{AMA}-{\text{M}}_{2}^{**}$	47	9	6	15 (31.91%)	PBC, IBD, pulmonary cancer, leukemia, fever, vomiting, physical examination
GP210 ^**^	18	0	3	3 (16.67%)	PBC, SS
SP100^**^	18	1	1	2 (11.11%)	PBC
SLA^**^	39	2	1	3 (7.69%)	PBC, HBV hepatitis, pulmonary infection

Only the information of RR positive patients having detection results of specific antibodies in PUMCH was analyzed. ASMA, antismooth muscle antibody; AMA, antimitochondrial antibody; AMA-M_2_, antimitochondrial antibody M_2_ subtype; CTD, connective tissue disease; SLE, systemic lupus erythematosus; APS, antiphospholipid syndrome; SSc, systemic sclerosis; SS, Sjogren’s syndrome; PBC, primary biliary cirrhosis; RA, rheumatoid arthritis; GBS, Guillain-Barrés syndrome; HA, hemolytic anemia; IBD, Inflammatory bowel disease; “*” means using indirect immunofluorescence. “**” means using chemiluminescence immunoassay (CLIA).

## Discussion

The IIF assay using HEp-2 cell substrate referred to as the unique ANA-screen assay is especially for patients suspected of autoimmune diseases (28). The RR pattern (AC-23) is a unique rod ring structure in the cytoplasm (29),which is easy to be recognized. Some reports showed that under special conditions, cytidine triphosphate synthase (CTPS) and IMPDH2 which played an important role in the cytidine and guanine nucleotide biosynthesis pathways, could aggregate into structures of RR. More specifically, RR structures are likely to form when the GTP/CTP pathways are inhibited by CTPS or IMPDH2 inhibitors such as 6-diazo-5-oxo-L-norleucine (DON), RBV, and mycophenolic acid (MPA) (17). Some studies indicated that IMPDH was the main autoantigen of anti-RR and the main component of RR structures, while complexes comprising RR structure and HCV proteins maybe facilitate the formation of anti-RR (30). Moreover, other unknown factors such as the combination of HCV and IFN/RBV may provide an autoimmune environment to induce the anti-RR response (31). Thus, the RR pattern is usually observed in hepatitis C. According to previous studies, about 9.2% to 70% of patients with hepatitis C could develop anti-RR antibodies after antiviral treatment with IFN or RBV (32–34), and the titer of the RR pattern would be gradually decreased and even lost with the end of treatment (35). This seems to indicate that anti-RR is a drug-induced autoantibody. However, recently some scholars also have found that anti-RR antibodies can exist in patients with hepatitis C before antiviral treatment or non-hepatitis C patients, patients with the therapy of other drugs, and even healthy people (36–38). The interaction between HCV protein and RR structure may not be necessary. At present, the correlation between anti-RR and demographic, clinical, or virological characteristics is unclear.

Our study retrospectively analyzes the ANA IIF results of 199,349 cases from two centers and finds that the prevalence of the RR pattern is rare in the Chinese population, which is only 0.16% in PUMCH and 0.23% in IMPH among the tested samples. The prevalence of ANA positive is 34.84% in PUMCH and 44.73% in IMPH. This is similar to the results of a multicenter study which showed that HEp-2 IFA positive rates range from 11.6-82.0% with a median of 28.5% (39). Although the determination of ANA by IIF using HEp-2 cell substrate is recommended as the gold standard, HEp-2 slides from different companies may show very different fluorescent patterns, and even some manufacturers’ reagents cannot show the RR pattern (30). This may be an important factor leading to the great difference in the clinical significance of the RR pattern in different countries or regions. In our research, the positive rates of RR pattern and ANA-positive in IMPH are both higher than that in PUMCH. The reason for this phenomenon may be that although female patients in PUMCH are more than those in IMPH, patients in IMPH are older than that in PUMCH (**Supplementary Table 1**), whereas higher ANA prevalence in older adults was also reported (40). Previous studies also recognized some potential differences of RR patterns between nationalities or ethnic groups (30). There are mainly Mongolian patients in IMPH, while there are more Han patients in PUMCH. It is reported that the ANA positive rate was higher in Tibetans, Huis, and Mongolians than in Han Chinese (39). In addition, regional differences in lifestyle, the level of culture and education, medical treatment and economic development, etc. will cause the difference in patients from the two centers. However, the positive rate of the RR pattern in ANA positive cases showed no difference between both centers. Anti-RR antibodies mainly appear in adults (≥ 41 years old), and the ratio of males to females was 1/1.3 and 1/1.5. By analyzing the patients with the RR pattern, we observed that fluorescence patterns of patients with RR pattern are mainly the isolated RR pattern, which was different from the results reported by Zhang L. et al (38) where the mixture of the speckled pattern was the most common. But in different diseases, the distribution of RR patterns was different, such as most healthy people with the isolated RR pattern, while CTDs patients mainly with the complex RR pattern, which may be because autoantibodies were much more common in CTDs than that in other diseases (41). In PUCMH, the low titer of 1:80 is more common, whereas no difference existed in titers of 1:80, 1:160, and 1:320 in IMPH. Cases with the titer of 1:640 were rare in both centers.

In cases with the RR pattern, AIDs (19.48%) are the most common in PUMCH, and nephropathy diseases are the most common in IMPH. The main reason for this difference is that the situation of disease distribution in the two centers is different, and there are more AIDs in PUMCH, while nephropathy diseases are the main in IMPH. AIDs were mainly presented as complex RR patterns, and other diseases were mainly presented as isolated patterns. Our study indicates that the RR pattern can exist in a variety of other diseases, such as AIDs, pulmonary diseases, nephropathy diseases, and even in healthy people. It is similar to a recent report from western China (38). This shows that the RR pattern is not only related to the antiviral treatment, but also possibly a consequence of other factors such as alterations in immune regulation caused by hepatitis or by autoimmune diseases, and even unknown environmental or genetic factors in different populations (23, 30). Interestingly, our study found that hepatic disease had the highest prevalence of RR pattern in PUMCH, and the prevalence of RR pattern for PBC and AIH is also higher than that in other AIDs. It is reported that the appearance of anti-RR may be a manifestation of metabolic disorders, while, the liver is the most important organ involved in metabolism (38). In PBC and AIH, the progression of the disease is related to the systematic increase of autoreactive antibodies and the possible massive infiltration of autoreactive lymphocytes in the liver (42). While IMPDH II is necessary for *de novo* purine synthesis in activated lymphocytes, it is also the main autoantigen of anti-RR and the main component of RR structures (30, 43). This may be why we observed a higher incidence of RR in hepatic diseases. Nevertheless, we did not find this trend in the data of IMPH, perhaps because of the limited sample size or incomplete clinical information.

Due to the incomplete clinical data of IMPH, the analysis of specific antibodies is only for some patients with the RR pattern in PUMCH. Secondly, this study is a retrospective study of a non-selected large-scale consecutive laboratory cohort over a period of time in both centers, and not every patient will receive specific antibody testing. Therefore, only the information of RR positive patients having results of detecting specific antibodies in PUMCH is analyzed. Of 128 patients with RR pattern having tested the ANA spectrum, specific antibodies were most common in patients with AIDs. The presence of the RR pattern linked with AIDs may be explained by alterations in immune regulation caused by autoimmunity in a particular genetic background, and AIDs patients are more likely to detect specific antibodies. Our retrospective analysis found that anti-Ro52 (27, 21.09%) is the most common target antibody in the ANA spectrum for RR positive cases. That may be because anti-Ro52 is a frequent autoantibody in AIDs, and is also described in some non-autoimmune disorders and a wide range of inflammatory disorders including malignant diseases, as well as in healthy controls (44). It was reported that there was a close relationship between anti-RR and anti-histone (30), but we have not confirmed the correlation between RR pattern and specific antibodies. In our study, we find that the RR pattern can be accompanied by a variety of autoantibodies, such as anti-RNP/Sm, anti-SSA, anti-AMA-M2, anti-GP210, anti-SP100, anti-SLA, etc. There have been similar reports that the RR pattern could be accompanied by antibodies related to autoimmune hepatitis (19, 45). As our study found, complex RR patterns were the most common in AIDs. While generally, ANA patterns corresponding to anti-AMA-M_2_, anti-GP210, and anti-SP100 were cytoplasmic reticular or mitochondria-like pattern, membranous pattern, and nuclear dots pattern, respectively. According to previously studies (46, 47), the most specific ANA patterns in PBC were the so-called Cytoplasmic reticular/AMA, rimlike/membranous, and multiple nuclear dots. Similarly, in autoimmune hepatitis patients, the most disease-specific ANA pattern was the “homogenous” one, as previously reported (48, 49).

Some scholars believe that due to the RR structure presenting in the pancreas and spleen (50), when damage to these organs are damaged, it will also induce the production of anti-RR even in the absence of hepatitis C infection or the use of drugs that induce RR structures (51). In highly proliferative cells, due to the increased demand for GTP nucleotides, it usually excites the RR structure assembly which will induce IMPDH to be a hyperactive state (52). A recent study has shown that cytidine triphosphate synthase (CTPS), which catalyzes the rate-limiting step in the *de novo* CTP synthetic pathway in which UTP is converted into CTP, also assembles into RR structures (53). All of them provide a possibility that screening for the presence of the RR pattern can be used to analyze the metabolic status and prognosis of tumors (54). Unfortunately, we did not observe whether RR pattern is related to tumors in our study, which may be because our clinical data are not detailed enough.

This study is a retrospective study of a non-selected large-scale consecutive laboratory cohort. Whereas, due to the imperfection of some research data, it has certain limitations in our study. First, it is not sure whether all patients with the RR pattern are treated by interferon. In the study, AIDs cases with the RR pattern were up to 19.48% in PUMCH. Moreover, it had been proved that the titer of the RR pattern would gradually decrease or disappear after stopping interferon therapy. Thus, these patients with AIDs were unlikely to receive interferon treatment. Secondly, although the RR pattern is ranked notifiable fluorescence pattern in first grade, the clinical significance of determining the RR pattern maybe need further research. This study mainly focuses on the distribution of the RR pattern in different diseases, which will be conducive to screening the RR pattern in a wide range of patients and pathological settings, however, its specific mechanism remains unclear. So, in order to give full play to the value of determining ANA in the clinical diagnosis and treatment, it is necessary for us to further study the clinical significance and mechanism of the RR pattern.

## Conclusion

In conclusion, this study systematically analyzed the clinical characteristics of the RR pattern in two centers of China and found the RR pattern has a low prevalence in ANAs test samples and varies in different nationalities and regions. The RR pattern exists in not only hepatitis C infection, but also in others (such as AIDs, hepatic cirrhosis, renal insufficiency, dermatosis, endocrine diseases, hematological diseases, etc.), and even in healthy people. Meanwhile, the prevalence of the RR pattern in hepatic disease may be higher than that in others.

## Data availability statement

The raw data supporting the conclusions of this article will be made available by the authors, without undue reservation.

## Ethics statement

The studies involving human participants were reviewed and approved by the ethics committee at Peking Union Medical College Hospital and fulfilled the ethical guidelines of the declaration of Helsinki (NO: S-478). Written informed consent to participate in this study was provided by the participants’ legal guardian/next of kin.

## Author contributions

CH and XZ conceived and designed the study. JM, GY, SL, YL, YB, CD, NS, ML conducted the clinical data extraction and literature search. JM and GY analyzed the data and wrote the draft of the manuscript. All authors critically revised the manuscript and approved the submission of the final version of the manuscript.

## Funding

This study was supported by the National Key Research and Development Program of China (2019YFC0840603), The National Natural Science Foundation of China (81771780), and The National High Level Hospital Clinical Research Funding (2022-PUMCH-A-039).

## Conflict of interest

The authors declare that the research was conducted in the absence of any commercial or financial relationships that could be construed as a potential conflict of interest.

## Publisher’s note

All claims expressed in this article are solely those of the authors and do not necessarily represent those of their affiliated organizations, or those of the publisher, the editors and the reviewers. Any product that may be evaluated in this article, or claim that may be made by its manufacturer, is not guaranteed or endorsed by the publisher.
