# Supplementary material for: The clinical value of indirect immunofluorescence for screening anti-rods and rings antibodies: A retrospective study of two centers in China
Source: Front Immunol. 2022 Sep 27;13:1007257. doi: 10.3389/fimmu.2022.1007257 (PMC9552219; doi:10.3389/fimmu.2022.1007257)
Supplement: Supplementary file 1 [file Table_1.docx]

**Supplementary Table S1. The demographic characteristics of the two centers**

| **Hospital** |  | **PUMCH** | **IMPH** | ***P value*** |
| --- | --- | --- | --- | --- |
| Case |  | 169891 | 29458 | — |
| M: F |  | 1: 2 | 1: 1.7 | 0.000 |
| Median age (Male) |  | 46(32-58) | 57(42-68) | 0.000 |
| Median age (Female) |  | 43(31-55) | 53(38-64) | 0.000 |
| Median age |  | 44(31-56) | 54(39-65) | 0.000 |

PUMCH: Peking Union Medical College Hospital. IMPH: Inner Mongolia People’s Hospital

The "*P value* " indicated the comparison of results between the two hospitals.

**Supplementary Table S2. Types of diseases**

| **Diseases**^*^ | **Inclusion** |
| --- | --- |
| Other pulmonary diseases | pulmonary shadow, pulmonary fibrosis, bronchial disease, pulmonary nodules, COPD, etc. |
| Other nephropathy diseases | hematuresis, nephritis, nephrotic syndrome, renal tubular acidosis, urinary system infection, etc. |
| Endocrine diseases | hypertension, diabetes Mellitus, thyroid disease, Cushing syndrome, etc. |
| Dermatosis | urticaria, vitiligo, dermatitis, rash, psoriasis, alopecia, etc. |
| Arthropathy | joint pain and arthritis. |
| Hematological diseases | leukemia, leukopenia, thrombocytopenia, anemia, etc. |
| Undefined diseases | ascites, abdominal infection, gastroenteritis, coronary heart disease, cardiopathy, ventricular failure, arrhythmia, cerebrovascular disease, cerebritis, etc. |

**Supplementary Table S3 The prevalence of RR pattern in patients**

| **Diseases** | **PUMCH** | | |  | **IMPH** | | |
| --- | --- | --- | --- | --- | --- | --- | --- |
|  | **n** | **(%)** | **Total** |  | **n** | **(%)** | **Total** |
| **Autoimmune diseases** | 51 | 0.14 | 35939 |  | 5 | 0.27 | 1878 |
| CTD | 13 | 0.16 | 8197 |  | 0 | 0.00 | 363 |
| RA | 12 | 0.23 | 5236 |  | 3 | 0.44 | 680 |
| SSc | 8 | 0.22 | 3709 |  | 0 | 0.00 | 23 |
| PBC | 4 | 0.85 | 473 |  | 0 | 0.00 | 14 |
| AIH | 1 | 0.65 | 153 |  | 0 | 0.00 | 50 |
| APS | 1 | 0.09 | 1134 |  | 0 | 0.00 | 21 |
| SLE | 4 | 0.04 | 11005 |  | 1 | 0.23 | 434 |
| Systemic vasculitis | 4 | 0.24 | 1650 |  | 1 | 1.19 | 84 |
| SS | 4 | 0.10 | 4113 |  | 0 | 0.00 | 196 |
| UC | 1 | 0.37 | 269 |  | 0 | 0.00 | 13 |
| **Pulmonary diseases** | 26 | 0.34 | 12891 |  | 18 | 0.73 | 1515 |
| Pulmonary interstitial diseases | 7 | 0.38 | 6023 |  | 5 | 1.17 | 171 |
| Neoplasm | 5 | 1.77 | 339 |  | 1 | 3.03 | 33 |
| Infection | 5 | 0.28 | 1804 |  | 2 | 0.27 | 366 |
| Others* | 9 | 0.19 | 4725 |  | 10 | 0.74 | 945 |
| **Nephropathy** | 44 | 0.13 | 19304 |  | 12 | 0.32 | 5618 |
| Renal insufficiency | 23 | 0.18 | 3911 |  | 2 | 0.44 | 1139 |
| Renal failure | 5 | 0.39 | 1526 |  | 1 | 0.67 | 447 |
| Proteinuria | 10 | 0.13 | 3962 |  | 1 | 0.45 | 442 |
| Others* | 6 | 0.08 | 9905 |  | 8 | 0.22 | 3590 |
| **Hepatic diseases** | 14 | 0.60 | 2320 |  | 2 | 0.20 | 997 |
| Hepatic dysfunction | 10 | 0.79 | 1266 |  | 0 | 0.00 | 579 |
| Hepatic cirrhosis | 2 | 1.05 | 191 |  | 1 | 0.39 | 259 |
| Hepatitis B | 1 | 0.57 | 175 |  | 0 | 0.00 | 55 |
| Hepatitis C | 0 | 0.00 | 17 |  | 1 | 14.29 | 7 |
| Hepatic tumor or cancer | 1 | 1.16 | 86 |  | 0 | 0.00 | 8 |
| **Other diseases** |  |  |  |  |  |  |  |
| Endocrine disease* | 19 | 0.16 | 11770 |  | 4 | 0.25 | 1631 |
| Dermatosis* | 20 | 0.11 | 17918 |  | 0 | 0.00 | 420 |
| Arthropathy* | 11 | 0.09 | 11765 |  | 5 | 0.14 | 3529 |
| Hematological diseases* | 15 | 0.14 | 10583 |  | 4 | 0.24 | 1668 |
| Fever | 13 | 0.20 | 6451 |  | 1 | 0.14 | 694 |

PUMCH: Peking Union Medical College Hospital. IMPH: Inner Mongolia People’s Hospital

Abbreviations: CTD: connective tissue disease. RA: rheumatoid arthritis. SSc: systemic sclerosis. PBC: primary biliary cirrhosis. AIH: autoimmune hepatitis. APS: antiphospholipid syndrome. SLE: systemic lupus erythematosus. SS: Sjogren’s syndrome. UC: ulcerative colitis.The "%" indicated the percentage refers to the proportion of positive cases of this disease.
